# Therapeutic Potentials of Selected Antihypertensive Agents and Their Fixed-Dose Combinations Against Trastuzumab-Mediated Cardiotoxicity

**DOI:** 10.3389/fphar.2020.610331

**Published:** 2021-03-04

**Authors:** Olufunke Esan Olorundare, Adejuwon Adewale Adeneye, Akinyele Olubiyi Akinsola, Abayomi Mayowa Ajayi, Olalekan Ayodele Agede, Sunday Sokunle Soyemi, Alban Ikenna Mgbehoma, Ikechukwu Innocent Okoye, Ralph M. Albrecht, James Mukasa Ntambi, Peter Anthony Crooks

**Affiliations:** ^1^ Department of Pharmacology and Therapeutics, Faculty of Basic Clinical Sciences, College of Health Sciences, University of Ilorin, Ilorin, Nigeria; ^2^ Department of Pharmacology, Therapeutics and Toxicology, Faculty of Basic Clinical Sciences, Lagos State University College of Medicine, Ikeja, Nigeria; ^3^ Department of Pharmacology and Therapeutics, Faculty of Basic Medical Sciences, University of Ibadan, Ibadan, Nigeria; ^4^ Department of Pathology and Forensic Medicine, Faculty of Basic Clinical Sciences, Lagos State University College of Medicine, Ikeja, Nigeria; ^5^ Department of Pathology and Forensic Medicine, Lagos State University Teaching Hospital, Ikeja, Nigeria; ^6^ Department of Oral Pathology and Medicine, Faculty of Dentistry, Lagos State University College of Medicine, Ikeja, Nigeria; ^7^ Department of Animal Sciences, University of Wisconsin, Madison, WI, United States; ^8^ Department of Nutritional Sciences, College of Agricultural and Life Sciences, University of Wisconsin, Madison, Madison, WI, United States; ^9^ Department of Pharmaceutical Sciences, College of Pharmacy, University of Arkansas for Medical Sciences, Little Rock, AR, United States

**Keywords:** Wistar rats, fixed-dose antihypertensive combinations, oxidative stress markers, cardiac injury biomarkers, trastuzumab cardiotoxicity

## Abstract

Trastuzumab (*TZM*) is useful in the clinical management of HER2-positive metastatic breast, gastric, and colorectal carcinoma but has been limited by its off-target cardiotoxicity. This study investigates the therapeutic potentials of 0.25 mg/kg/day amlodipine, 0.035 mg/kg/day lisinopril, 5 mg/kg/day valsartan, and their fixed-dose combinations in *TZM*-intoxicated Wistar rats that were randomly allotted into 10 groups of 6 rats for each group. Group I rats were treated with 10 ml/kg/day sterile water orally and 1 ml/kg/day sterile water intraperitoneally; Groups II, III, and IV rats were orally gavaged with 5 mg/kg/day valsartan and 1 ml/kg/day sterile water intraperitoneally, 0.25 mg/kg/day amlodipine and 1 ml/kg/day sterile water via the intraperitoneal route, 0.035 mg/kg/day lisinopril and 1 ml/kg/day sterile water administered intraperitoneally, respectively. Group V rats were orally treated with 10 ml/kg/day of sterile water prior to intraperitoneal administration of 2.25 mg/kg/day of *TZM*. Groups VI–VIII rats were equally pretreated with 5 mg/kg/day valsartan, 0.25 mg/kg/day amlodipine, and 0.035 mg/kg/day lisinopril before intraperitoneal 2.25 mg/kg/day *TZM* treatment, respectively; Groups IX and X rats were orally pretreated with the fixed-dose combinations of 0.25 mg/kg/day amlodipine +0.035 mg/kg/day lisinopril and 5 mg/kg/day valsartan +0.035 mg/kg/day lisinopril, respectively, before *TZM* treatment. Cardiac injury and tissue oxidative stress markers, complete lipids profile, histopathological, and immunohistochemical assays were the evaluating endpoints. Results showed that repeated *TZM* treatments caused profound increases in the serum TG and VLDL-c levels, serum cTnI and LDH levels, and cardiac tissue caspase-3 and -9 levels but decreased BCL-2 expression. *TZM* also profoundly attenuated CAT, SOD, GST and GPx activities, and increased MDA levels in the treated tissues. In addition, *TZM* cardiotoxicity was characterized by marked vascular and cardiomyocyte congestion and coronary artery microthrombi formation. However, the altered biochemical, histopathological, and immunohistochemical changes were reversed with amlodipine, lisinopril, valsartan, and fixed-dose combinations, although fixed-dose valsartan/lisinopril combination was further associated with hyperlipidemia and increased AI and CRI values and coronary artery cartilaginous metaplasia. Thus, the promising therapeutic potentials of amlodipine, lisinopril, valsartan and their fixed-dose combinations in the management of *TZM* cardiotoxicity, majorly mediated *via* antiapoptotic and oxidative stress inhibition mechanisms were unveiled through this study.

## Introduction

Cancer remains a major public health issue all around the world and is presently considered the second leading cause of death globally, accounting for an estimated 9.6 million deaths or one in six deaths with approximately 70% of these deaths occurring in low- and middle-income countries in the year 2018 (World Health Organization, 2018; Siegel et al., 2020). This figure has been predicted to increase to 19 million sufferers by the year 2030 (Corremans et al., 2019). However, cancer treatment prognosis has tremendously improved in recent times due to better diagnostic tools, earlier detection, and more effective therapeutic strategies including novel-targeted therapies, one of which is the monoclonal antibody, trastuzumab.

Trastuzumab (*TZM*) (sold under the brand names Herceptin®, Herzuma®, Ogivri®, etc.) is a recombinant DNA-derived, humanized mouse IgG kappa monoclonal antibody targeted against the subdomain IV of the extracellular region of human epidermal growth factor receptor 2 (HER2)-expressed tumors (Poon et al., 2013; Porta et al., 2015; Fang et al., 2020). *TZM* is one of the HER2-targeted monoclonal antibodies approved for the clinical management of HER2 overexpressing metastatic solid tumors such as breast and gastric cancers (Lameire, 2014; Porta et al., 2015), gastroesophageal adenocarcinoma (Blackwell et al., 2010; Poon et al., 2013), and salivary duct carcinoma (Gibo et al., 2019). *TZM* binds to the extracellular membrane domain of HER 2 to inhibit proliferation, survival and reversal of the phenotype of HER2/neu expressing tumor cells (Mandaliya et al., 2015). The exact cytotoxic actions of *TZM* are believed to be multimodal and these include inhibition of: signal transduction pathways, extracellular domain cleavage, and DNA repair, as well as decreased angiogenesis, induction of cell cycle arrest, and activation of antibody dependent cellular cytotoxicity (Poon et al., 2013; Lameire, 2014). The wide clinical application of *TZM* has profoundly improved the survival and recovery chances of patients with advanced HER2-positive breast and gastric cancers but has reportedly been limited by its cumulative and reversible off-target organ toxicities, most common of which is cardiotoxicity (Hidalgo et al., 2013; Onitilo et al., 2014; Mohan et al., 2018).

There are reports of the potential role of some classes of antihypertensive agents as effective modulators of anthracycline- and trastuzumab-induced cardiotoxicities (Rygiel, 2016). These classes of antihypertensive drugs include angiotensin-converting enzyme inhibitors (ACEIs), angiotensin receptor blockers (ARBs), and beta(β)_1_-adrenoreceptor blockers (Gujral et al., 2018; Sharma et al., 2018; Guglin et al., 2019; Ma et al., 2019; Guo et al., 2020) in the prevention of anthracycline- and *TZM*-induced cardiotoxicities (Wittayanukorn et al., 2018; Blanter and Frishman, 2019; Brown et al., 2020). In addition, ranolazine, a new anti-ischemia drug and a specific inhibitor of late sodium current, has been reported to attenuate *TZM*-induced heart dysfunction by reducing reactive oxygen species (ROS) production (Riccio et al., 2018). However, despite the availability and clinical trials of these potential antidotes, their effectiveness in ameliorating *TZM*-induced cardiotoxicity is still debatable. Therefore, this study is aimed at evaluating the possible therapeutic potential of amlodipine, lisinopril, and valsartan (being prototype of classical long-acting dihydropyridine calcium channel blockers, angiotensin-converting enzyme inhibitors, and angiotensin receptor blockers, respectively) and their fixed-dose [(amlodipine + lisinopril) and (valsartan + lisinopril)] combinations in acute *TZM*-induced cardiotoxicity in Wistar rats for the sole reason of therapeutic drug repurposing. In doing this, effects of the oral pretreatments with these drugs and their fixed-dose combinations on biochemical (cardiac enzyme markers, complete lipids profile, cardiac oxidative stress markers, and markers of apoptosis), histopathological, and immunohistochemical endpoints were evaluated in *TZM*-induced cardiotoxicity. The choice of the drug fixed-dose combinations was based on their reported high efficacies in regulating blood pressure and preventing complications in hypertensive patients (Naidu et al., 2000; Locatelli et al., 2002; Menne et al., 2008; Alhamdani, 2009; Bubnova et al., 2019).

## Materials and Methods

### Drugs and Chemicals

Drugs used include trastuzumab injection with an accompanying sterile water vial (Biocon CANMab 440 mg lyophilized powder™, Biocon Limited, Km 34 Tumkur Road, T-Bengur, Nelamangala Taluk, Bangalore-56 123, India), amlodipine besylate (Pfizer Norvasc 5 mg™, R-Pharm Germany GmbH, Heinrich-Mack-Str. 35, 89257 Illertissen, Germany), lisinopril dihydrate (Zestril 5 mg™, AstraZeneca United Kingdom Limited, Macclesfield, Cheshire, SK10 2NA, United Kingdom), and valsartan (Norvatis Diovan 160™, Norvatis Pharma AG, Basel, Switzerland). Chemicals such as hydrochloric acid (HCl), thiobarbituric acid (TBA), 1,2-dichloro-4-nitrobenzene, trichloroacetic acid (TCA), sodium hydroxide, xylenol orange (XO), potassium hydroxide, reduced glutathione (GSH), and hydrogen peroxide (H_2_O_2_) were purchased from Sigma-Aldrich Chemical Co., St. Louis, MO, USA. All other chemicals were of analytical grade and were purchased from ThermoFisher Scientific, Cambridge, Massachusetts, USA. Other reagents and analytical test kits used include: Thermo Scientific Pierce™ 36000 Peroxidase IHC Detection Kits (Pierce Biotechnology, Rockford, IL 61105, USA), caspase 3 monoclonal antibody [(3CSP01 (7.1.44), catalog no. MA5-11516, ThermoFisher Scientific, Cambridge, Massachusetts, USA), BCL-2 monoclonal antibody (Catalog no. MA5-11757, ThermoFisher Scientific, Cambridge, Massachusetts, USA), avidin/streptavidin–horseradish peroxidase (HRP) conjugate (Product catalog: SA100-01, Invitrogen Life Technologies, Carlsbard, CA 92008, USA), DAB (3,3’ diaminobenzidine) substrate working solution [Product catalog: 34001 (DAB), 34002 (DAB Substrate kit, Pierce Biotechnologies, Rockford, IL 61105, USA], dibutylphthalate polystyrene xylene (DPX) mountant (Sigma-Aldrich Inc., St. Louis, MO 63118, USA), lipid test kits (MAK043, MAK045, and TRO100) (Sigma-Aldrich Inc., St. Louis, MO 63118, USA), cardiac troponin-I ELISA kit (ab20001), and lactate dehydrogenase (LDH) assay kit (colorimetric) (ab102526) (Abcam Biotechnology Company, 1 Kendall Square, Suite B2304, Cambridge, MA 02139117, USA).

### Experimental Animals

Sixty (60) male Wistar albino rats aged 8–12 weeks old and weighing 170–190 g used for the experiment were procured from the Animal House of the Lagos State University College of Medicine, Ikeja, Lagos State, Nigeria, after an ethical approval (UERC approval number: UERC/ASN/2020/2027) was obtained from the University of Ilorin Ethical Review Committee for Postgraduate Research. The rats were handled in accordance with international principles guiding the use and handling of experimental animals (National Research Council (US), 2011; Committee for the Update of the Guide for the Care and Use of Laboratory Animals). The experimental rats were placed on standard rat feed (Ladokun Feeds, Ibadan, Oyo State, Nigeria) and potable water *ad libitum* and maintained under standard laboratory conditions (ambient temperature: 23–26°C, 55 ± 5% humidity, and 12/12-h alternating light and dark periodicity).

### Body Weight Measurement

The body weights of rats were taken on days 1 and 7 of the experiment using a digital rodent weighing scale (®Virgo Electronic Compact Scale, New Delhi, India). The obtained values were expressed in grams (g).

### Experimental Induction of *TZM*-Induced Cardiotoxicity and Drug Treatments of Rats

Before the experiment started, rats were randomly allotted into 10 groups of 6 rats per group such that differences in-between and within groups of their weights were not more than ±20% of the average weight of the sample population of rats used for the study. However, the choice of the therapeutic doses of amlodipine, lisinopril, valsartan and their fixed-dose combinations were made based on the results of the orientation studies earlier conducted by us. The dose of *TZM* adopted was as described by Poon et al., (2013) and Riccio et al., (2018).

In this experimental repeated dose model, Group I rats served as untreated control and were orally pretreated with 10 ml/kg/day of sterile water before *i. p.* treatment with 1 ml/kg/day of sterile water for 7 days (Table 1). Groups II, III, and IV rats were orally treated with 5 mg/kg/day of valsartan, 0.25 mg/kg/day of amlodipine, and 0.035 mg/kg/day of lisinopril, all dissolved in sterile water 3 h before *i. p.* treatment with 1 ml/kg/day of sterile water each day for 7 days, respectively. Group V rats were orally pretreated with 10 ml/kg/day of sterile water 3 h prior to *i. p.* 2.25 mg/kg/day of *TZM* for 7 days (Table 1). Groups VI–VIII rats were equally pretreated with 5 mg/kg/day of valsartan, 0.25 mg/kg/day of amlodipine, and 0.035 mg/kg/day of lisinopril, all dissolved in sterile water 3 h before *i. p.* treatment with 2.25 mg/kg of *TZM* for 7 days, respectively (Table 1). Groups IX and X rats were orally pretreated with the fixed-dose combinations of 0.25 mg/kg/day amlodipine +0.035 mg/kg/day lisinopril in dissolved in sterile water and 5 mg/kg/day valsartan +0.035 mg/kg/day lisinopril in dissolved in sterile water, respectively, 3 h before *i. p.* treatment with 2.25 mg/kg of *TZM* dissolved in sterile water for 7 days (Table 1).

**TABLE 1:** Drug treatment of rats.

Groups	Treatments
Group I	10 ml/kg/day of sterile water given *p.o.* + 1 ml/kg/day of sterile water given *i.p.* for 7 days
Group II	5 mg/kg/day of valsartan in sterile given *p.o.* + 1 ml/kg/day of sterile water given *i.p.* for 7 days
Group III	0.25 mg/kg/day of amlodipine in sterile water given *p.o.* + 1 ml/kg of sterile water given *i.p.* for 7 days
Group IV	0.035 mg/kg/day of lisinopril dissolved in sterile water given *p.o.* + 1 ml/kg of sterile water given *i.p.* for 7 days
Group V	10 ml/kg/day of sterile water given *p.o.* + 2.25 mg/kg/day of trastuzumab given *i.p.* for 7 days
Group VI	5 mg/kg/day of valsartan in sterile water given *p.o.* + 2.25 mg/kg of trastuzumab given *i.p.* for 7 days
Group VII	0.25 mg/kg/day of amlodipine in sterile water given *p.o.* + 2.25 mg/kg/day of trastuzumab given *i.p.* for 7 days
Group VIII	0.035 mg/kg/day of lisinopril dissolved in sterile water given *p.o.* + 2.25 mg/kg/day of trastuzumab given *i.p.* for 7 days
Group IX	0.25 mg/kg/day of amlodipine +0.035 mg/kg/day of lisinopril dissolved in sterile water and given *p.o.* for 7days +2.25 mg/kg/day of trastuzumab given *i.p.* for 7 days
Group X	5 mg/kg/day of valsartan +0.035 mg/kg/day of lisinopril dissolved in sterile water and given *p.o.* + 2.25 mg/kg/day of trastuzumab given *i.p.* for 7 days

### Blood Sample Collection

24 h following the last *TZM* dose, rats were sacrificed under inhaled light diethyl ether anesthesia. All the animals were subjected to an overnight fast before they were sacrificed. Blood samples were collected into plain blood sample bottles directly from the heart chambers with fine needle (21G size) mounted on a 5 ml syringe (Hangzhou Longde Medical Products Co. Ltd., Hangzhou, China) without causing damage to the heart tissue.

### Harvesting and Weighing of Selected Vital Body Organs

Selected vital organs such as the heart, liver, kidneys, and lungs of treated rats were identified, harvested *en bloc*, and weighed on a digital weighing scale.

### Measurement of Serum Cardiac Enzyme Markers and Lipids

Blood samples obtained were collected into a 10 ml plain sample bottle and allowed to clot and then centrifuged at 5,000 rpm for 5 min in order to separate out clear sera which were analyzed for the following biochemical parameters: serum cardiac injury markers [cardiac troponin I (*cTnI*) and lactate dehydrogenase (LDH)] and lipids (TG, TC, HDL-c, and LDL-c) using standard procedures described in the accompanying leaflets in the commercial test kits.

### Calculation of Atherogenic Index of Plasma (AIP) and Coronary Artery Index (CRI)

AIP value was calculated as: [log_10_ (TG) (mg/dl) ÷ HDL-c (mg/dl) (Dobiasova and Frohlich, 2001; Dobiasova, 2004; Nansseu et al., 2016), while CRI was calculated as: TC (mg/dl) ÷ HDL-c (mg/dl) (Alladi and Shanmugasundaram, 1989).

### Determination of Antioxidant Activities in the Rat Cardiac Tissues

Following the humane sacrifice of treated rats under light inhaled diethyl ether, rat heart was identified and gently dissected out *en bloc* and carefully divided into two halves (each consisting of the atrium and ventricle) using a new surgical blade. The left half of the heart was briskly rinsed in ice cold 1.15% KCl solution in order to preserve the oxidative enzyme activities of the heart before being placed in a clean sample bottle which itself was in an ice pack–filled cooler. This was done to prevent the breakdown of the oxidative stress enzymes in these organs.

### Determination of Superoxide Dismutase (SOD) Activities in the Cardiac Tissues

Enzyme activity of SOD was determined by its ability to inhibit the auto-oxidation of epinephrine by the increase in absorbance at 480 nm as described by Paoletti et al., (1986). SOD activity was subsequently calculated by measuring the change in absorbance at 480 nm for 5 min.

### Determination of Catalase (CAT) Activity in the Cardiac Tissue

Cardiac tissue CAT activity was determined using the method described by Hadwan (2018), and the specific activity of CAT was expressed as U/ml.

### Determination of Cardiac Tissue Reduced Glutathione (GSH), Glutathione Peroxidase (GPx), and Glutathione-*S*-Transferase (GST) Activities

GSH content in the cardiac tissues was estimated using the method earlier described by Rahman et al., (2006). To the cardiac homogenate, 10% TCA was added and centrifuged. 1 ml of the supernatant was treated with 0.5 ml of Elman’s reagent (19.8 mg of 5,5-dithiobisnitro benzoic acid (DTNB) in 100 ml of 0.1% sodium nitrate) and 3.0 ml of phosphate buffer (0.2 M, pH 8.0) and its absorbance was read at 412 nm. Similarly, GPx and GST activities were determined using the method of Vontas et al., (2000).

### Determination of Cardiac Tissue Malondialdehyde (MDA) Contents

1 ml of supernatant was added to 2 ml of (1:1:1 ratio) TCA–TBA–HCl reagent (composition: thiobarbituric acid 0.37%, 0.24 N HCl, and 15% TCA), boiled at 100°C for 15 min, allowed to cool, and then centrifuged at 3,000 rpm for 10 min so as to remove the flocculent material. The supernatant was removed and its absorbance was read at 532 nm against a blank. MDA was calculated using the molar extinction for MDA–TBA complex of 1.56 × 10⁵ m^−1^ cm^−1^ as described by Buege and Aust (1978).

### Histopathologic Assessment of the Heart Tissues

Using the remaining equally divided harvested heart, the right halves of the six randomly selected rats from each treatment and control groups were subjected to histopathological examinations, the right ventricle being the most susceptible to *TZM* toxicity of the heart chambers (Calleja et al., 2015; Barthur et al., 2017; Jahangir et al., 2020). After rinsing in normal saline, the dissected right half of was preserved in 10% formosaline before it was completely dehydrated in absolute ethanol. It was then embedded in paraffin wax blocks. 4–5 μm thick sections of the tissue were prepared from the paraffin block before staining with hematoxylin–eosin. Thereafter, these were examined under a photomicroscope (Model N-400ME, CEL-TECH Diagnostics, Hamburg, Germany) connected with a host computer. Sections were illuminated with white light from a 12 V halogen lamp (100 W) after filtering with a 520-nm monochromatic filter. The slides were examined for associated histopathological lesions (Slaoui and Fiette, 2011).

### Enzyme Linked Immunosorbent Assay (ELISA) for Determination of Cardiac Tissue Caspase-3 and Caspase-9

Cardiac tissue levels of caspase-3 and caspase-9 were determined using the commercial Enzyme linked immunosorbent assay (ELISA) kits (Wuhan Elabscience Biotechnology Company Limited, No. 1 Shizishan Street, Hongshan District, Wuhan, Hubei, China) following the Manufacturers' instruction.

### Immunohistochemical Studies of Heart Tissues for Caspase-3 and BCL-2 Levels and Expressions

#### Assessment of Caspase-3 and BCL-2 Levels and Expressions

This was done following the Manufacturer’s procedures contained in the product leaflets accompanying the test kits. Small section of the right heart ventricle was trimmed off and was fixed in 10% formalin neutral buffer for histological processing and paraffin embedding. 4–5-μm thin sections of the tissue were micro-sectioned, floated, and mounted on charged glass slides. The slides were labeled, arranged in racks, and placed in oven at 50–60°C for 20–30 min to melt excess paraffin. The slides containing heart tissues were further deparaffinized and prepared for heat-induced antigen retrieval in citrate buffer solution (10 mM citric acid, pH 6.0). The staining was performed using Thermo Scientific Pierce™ 36000 Peroxidase IHC Detection Kits with slight modification of the procedure. Endogenous peroxidase activity was quenched by incubating heart tissues for 30 min in Peroxidase Suppressor and washed three times in wash buffer. Blocking buffer was added to the slides and incubated for 30 min. Excess buffer was blotted from the tissue sections, before addition of primary antibodies: Caspase-3 monoclonal antibody, BCL-2 monoclonal antibody at a dilution of 1:100, and left overnight in a humidified chamber at 4°C. Afterward, slides were washed two times for 3 min with wash buffer. The tissue sections were treated with biotinylated secondary antibody and incubated for 30 min. The slides were washed thrice for 3 min each with wash buffer, treated and incubated with avidin/streptavidin–horseradish peroxidase conjugate for another 30 min, and washed three times for 3 min each with wash buffer. The tissues were incubated with metal-enhanced DAB (3,3′ diaminobenzidine) substrate working solution for 5 min for desired staining to achieve. The slides were rinsed with distilled water and drained. Adequate amount of Mayer’s hematoxylin stain was dropped on the slide to cover the entire tissue surface and incubated for 1–2 min at room temperature. Hematoxylin stain was then drained off and the slides washed several times with distilled water. The slides prepared were mounted with cover slips and dibutylphthalate polystyrene xylene (DPX) mountant for histology.

### Assessment of Caspase-9 Levels and Expressions

Similar procedure was adopted in the caspase-9 assay using the commercial ELISA kits (Wuhan Elabscience Biotechnology Company Limited, No. 1 Shizishan Street, Hongshan District, Wuhan, Hubei, China) following the Manufacturers’ instruction.

### Scoring-Based Stain Intensity

Photomicrographs were taken with AmScope MU900 9MP USB2.0 Microscope Digital Camera attached to Fisher Science Education™ 160-Series University/Laboratory Compound Microscope (Model: S2387, Fisher Scientific, California, United States). The images were quantified for staining intensity using Fiji (open source image processing package based on ImageJ) software (Varghese et al., 2014).

### Statistical Analysis

Data were presented as mean ± SD and mean ± SEM of six observations for the body weight/percentage body weight changes and biochemical parameters, respectively, while data for immunohistochemical analysis were expressed as mean ± SEM of four observations in duplicates. One-way ANOVA followed by Turkey’s *post hoc* test, on GraphPad Prism version 5, was used for statistical analysis of the data obtained. Statistical significance was considered at *p* < 0.05, *p* < 0.001, and *p* < 0.0001.

## Results

### Effect of Amlodipine, Lisinopril, Valsartan and Their Fixed-Dose Treatments on Body Weight Changes (% ∆bwt.)

Effects of repeated daily intraperitoneal injection with 2.25 mg/kg of *TZM* and oral pretreatments with ADP, LSP, VAL and the fixed-dose combinations of ADP + LSP and VAL + LSP, respectively, on the average body weight and % ∆bwt. of rats on days 1 and 7 are as indicated in Table 2. Oral pretreatments with ADP and LSP to normal rats resulted in significant (*p* < 0.001) reductions in % ∆bwt. when compared to Group I values. Similarly, *i. p. TZM* treatment and oral pretreatments with ADP, LSP, VAL and their combinations caused similar significant (*p* < 0.001) weight loss in *TZM*-intoxicated rats when compared to Group I rats (Table 2).

**TABLE 2:** Effect of repeated oral treatment with amlodipine, lisinopril, valsartan and their fixed-dose combinations on the average body weight and percentage body weight changes (% ∆bwt.) of *TZM*-treated rats.

**Groups**	**Body wt. on Day 1 (g)**	**Body wt. on Day 7 (g)**	**% ∆bwt.**
I	208.60 ± 32.22	223.20 ± 35.12	06.95 ± 05.03
II	203.00 ± 17.43	216.70 ± 18.64	06.79 ± 03.83
III	200.60 ± 28.18	207.10 ± 30.61	03.25 ± 02.76^b^
IV	206.10 ± 20.88	208.80 ± 18.82	02.12 ± 01.91^b−^
V	194.90 ± 11.56	195.30 ± 16.28	00.47 ± 10.60^b−^
VI	201.40 ± 16.36	204.40 ± 16.00	01.57 ± 03.85^b−^
VII	202.80 ± 15.36	209.30 ± 19.05	03.13 ± 02.99^b−^
VIII	204.60 ± 13.59	210.80 ± 06.87	02.98 ± 03.70^b−^
IX	207.00 ± 10.24	201.80 ± 13.34	01.80 ± 03.17^b−^
X	194.80 ± 20.82	198.90 ± 24.05	02.08 ± 03.63^b−^

^b−^ represents a significant decrease at p < 0.001 when compared to untreated normal control (Group I) and valsartan-treated rats (Group II).

Groups I: untreated normal control; Group II: 5 mg/kg/day valsartan; Group III: 0.25 mg/kg/day amlodipine; Group IV: 0.035 mg/kg/day lisinopril; Group V: untreated TZM control; Groups VI: 5 mg/kg/day valsartan +2.25 mg/kg/day TZM; Group VII: 0.25 mg/kg/day amlodipine +2.25 mg/kg/day TZM; Group VIII: 0.035 mg/kg/day lisinopril +2.25 mg/kg/day TZM; Groups IX: [0.25 mg/kg/day amlodipine +0.035 mg/kg/day lisinopril] + 2.25 mg/kg/day TZM; Group X: [5 mg/kg/day valsartan +0.035 mg/kg/day lisinopril] + 2.25 mg/kg/day TZM.

### Effect of Amlodipine, Lisinopril, Valsartan and their Fixed-Dose Combinations on the Relative Organ Weights of *TZM*-Treated Rats

*TZM* treatment did not cause any significant (*p* > 0.05) alterations in RHW, RLW, and RKW relative to Group I values (Table 3). Similar effect was recorded for *TZM*-intoxicated rats pretreated with ADP, LSP, VAL and their fixed-dose combinations (Table 3).

**TABLE 3:** Effect of repeated oral treatment with amlodipine, lisinopril, valsartan and their fixed-dose combinations on the relative organ weight of *TZM*-treated rats.

Group	Relative organ weight			
	Heart	Liver	Kidney	Lungs
I	0.33 ± 0.05	3.34 ± 0.56	0.55 ± 0.06	0.80 ± 0.09
II	0.34 ± 0.06	3.45 ± 0.77	0.56 ± 0.12	0.84 ± 0.20
III	0.31 ± 0.05	3.24 ± 0.35	0.62 ± 0.07	0.78 ± 0.19
IV	0.31 ± 0.06	3.20 ± 0.40	0.60 ± 0.05	0.77 ± 0.18
V	0.36 ± 0.06	3.24 ± 0.60	0.59 ± 0.09	0.83 ± 0.14
VI	0.28 ± 0.03	3.17 ± 0.34	0.63 ± 0.07	0.70 ± 0.08
VII	0.34 ± 0.03	3.30 ± 0.49	0.61 ± 0.10	0.74 ± 0.10
VIII	0.30 ± 0.03	3.28 ± 0.24	0.56 ± 0.06	0.70 ± 0.17
IX	0.35 ± 0.02	3.44 ± 0.38	0.58 ± 0.55	0.69 ± 0.08
X	0.35 ± 0.05	3.53 ± 0.62	0.59 ± 0.04	0.73 ± 0.15

Groups I: untreated normal control; Group II: 5 mg/kg/day valsartan; Group III: 0.25 mg/kg/day amlodipine; Group IV: 0.035 mg/kg/day lisinopril; Group V: untreated TZM control; Groups VI: 5 mg/kg/day valsartan +2.25 mg/kg/day TZM; Group VII: 0.25 mg/kg/day amlodipine +2.25 mg/kg/day TZM; Group VIII: 0.035 mg/kg/day lisinopril +2.25 mg/kg/day TZM; Groups IX: [0.25 mg/kg/day amlodipine +0.035 mg/kg/day lisinopril] + 2.25 mg/kg/day TZM; Group X: [5 mg/kg/day valsartan +0.035 mg/kg/day lisinopril] + 2.25 mg/kg/day TZM.

### Effect of Amlodipine, Lisinopril, Valsartan and Their Fixed-Dose Combinations on the Serum Lipids (TG, TC, HDL-c, LDL-c, and VLDL-c) Levels in *TZM*-Treated Rats

Repeated intraperitoneal *TZM* injections resulted in a significant (*p* < 0.05) increase in the serum TG levels when compared to untreated normal control values, while ADP, LSP, VAL and their fixed-dose combination pretreatments resulted in significant (*p* < 0.05) reduction in serum TG levels when compared to untreated *TZM*-intoxicated rats (Table 4). Although *TZM i. p.* treatment did not significantly (*p* > 0.05) alter the serum TC levels, but oral pretreatment with VAL significantly (*p* < 0.05) reduced serum TC levels while ADP + LSP and VAL + LSP significantly (*p* < 0.05) elevated serum TC levels in *TZM*-intoxicated rats (Table 4). Similarly, oral pretreatments with the fixed-dose ADP + LSP and VAL + LSP combinations induced significant (*p* < 0.05) increases in the serum LDL-c and VLDL-c levels in *TZM*-intoxicated rats.

**TABLE 4:** Effect of repeated oral administration of amlodipine, lisinopril, valsartan and their fixed-dose combinations on serum lipid profile of *TZM*-treated rats.

Groups	Serum lipids				
	TG (mmol/l)	TC (mmol/l)	HDL-c (mmol/l)	LDL-c (mmol/l)	VLDC-c (mmol/l)
I	0.71 ± 0.11	1.10 ± 0.21	0.45 ± 0.06	0.40 ± 0.11	0.32 ± 0.05
II	1.00 ± 0.15	1.66 ± 0.20^a+^	0.48 ± 0.08	0.73 ± 0.07	0.45 ± 0.07
III	0.86 ± 0.08	1.32 ± 0.08	0.39 ± 0.01	0.53 ± 0.09	0.39 ± 0.04
IV	1.02 ± 0.17	1.47 ± 0.12	0.42 ± 0.04	0.59 ± 0.10	0.46 ± 0.08
V	1.17 ± 0.21^a+^	1.20 ± 0.26	0.43 ± 0.06	0.49 ± 0.06	0.52 ± 0.08^a+^
VI	0.70 ± 0.05^a*^	1.05 ± 0.15^a*^	0.37 ± 0.04	0.52 ± 0.13	0.32 ± 0.02
VII	0.95 ± 0.25	1.22 ± 0.14	0.32 ± 0.02	0.47 ± 0.06	0.51 ± 0.10^a+^
VIII	0.85 ± 0.20^a*^	1.15 ± 0.16	0.33 ± 0.04	0.43 ± 0.06	0.38 ± 0.09
IX	0.90 ± 0.14^a*^	1.72 ± 0.13^a+,a^	0.40 ± 0.03	0.92 ± 0.15^b+,b^	0.41 ± 0.06
X	0.48 ± 0.03^c*^	1.67 ± 0.09^a+,a^	0.36 ± 0.02	1.10 ± 0.09^c+,c^	0.22 ± 0.01^a*^

^a+,^ ^b+,^ and ^c+^ represent significant increases at p < 0.05, p < 0.001, and p < 0.0001, respectively, when compared to untreated normal control (Group I) value, while ^a, b,^ and ^c^ represent significant increases at p < 0.05, p < 0.001, and p < 0.0001, respectively, when compared to untreated TZM-treated only (Group V) values, while ^a*, b*,^ and ^c*^ represent significant decreases at p < 0.05, p < 0.001, and p < 0.0001, respectively, when compared to untreated TZM-intoxicated only (Group V).

Groups I: untreated normal control; Group II: 5 mg/kg/day valsartan; Group III: 0.25 mg/kg/day amlodipine; Group IV: 0.035 mg/kg/day lisinopril; Group V: untreated TZM control; Groups VI: 5 mg/kg/day valsartan +2.25 mg/kg/day TZM; Group VII: 0.25 mg/kg/day amlodipine +2.25 mg/kg/day TZM; Group VIII: 0.035 mg/kg/day lisinopril +2.25 mg/kg/day TZM; Groups IX: [0.25 mg/kg/day amlodipine +0.035 mg/kg/day lisinopril] + 2.25 mg/kg/day TZM; Group X: [5 mg/kg/day valsartan +0.035 mg/kg/day lisinopril] + 2.25 mg/kg/day TZM.

### Amlodipine, Lisinopril, Valsartan and Their Fixed-Dose Combinations on the Atherogenic Index and Coronary Artery Disease Index of *TZM*-Treated Rats

Repeated intraperitoneal 2.25 mg/kg/day *TZM* injection for 7 days to treated rats resulted in a significant (*p* < 0.05) increase in AI without any significant (*p* > 0.05) alterations in the CRI values when compared to the untreated control (Group I) (Table 5). However, with ADP, LSP, and VAL pretreatments, there were significant (*p* < 0.05 and *p* < 0.001) decreases in the AI values but no significant (*p* > 0.05) alterations in CRI values when compared to the untreated *TZM* control (Group V) values (Table 5). Conversely, fixed-dose [(ADP + LSP) and (VAL + LSP)] combination pretreatments significantly (*p* < 0.05, *p* < 0.001, and *p* < 0.0001) increased the AI and CRI values with the higher values induced by the VAL + LSP combinations than untreated *TZM*-intoxicated (Group V) values (Table 5).

**TABLE 5:** Effect of amlodipine, lisinopril, valsartan and their fixed-dose combinations on atherogenic index of plasma (AIP) and coronary artery disease index (CRI) in *TZM*-intoxicated rats.

Treatment Groups	AIP	CRI
I	−0.01 ± 0.05	02.95 ± 0.14
II	0.06 ± 0.17^a−^	03.45 ± 0.11
III	0.09 ± 0.08^a−^	03.35 ± 0.13
IV	0.01 ± 0.17^a−^	03.53 ± 0.19
V	0.11 ± 0.03^a*^	03.43 ± 0.15
VI	−0.09 ± 0.09^c−^	02.83 ± 0.10
VII	0.09 ± 0.06^a−^	03.86 ± 0.56
VIII	−0.44 ± 0.13^c−^	03.42 ± 0.13
IX	0.22 ± 0.15^b+^	04.40 ± 0.40^a+^
X	03.19 ± 0.40^c+^	04.82 ± 0.52^a+^

^a*^ represents a significant increase at p < 0.05 when compared to untreated control (Group I); ^b+^ and ^c+^ represent significant increases at p < 0.001 and p < 0.0001, respectively, while ^a−^ and ^c−^ represent significant decreases at *p* < 0.05 and *p* < 0.0001, respectively, when compared to untreated TZM-intoxicated (Group V) values.

Groups I: untreated normal control; Group II: 5 mg/kg/day valsartan; Group III: 0.25 mg/kg/day amlodipine; Group IV: 0.035 mg/kg/day lisinopril; Group V: untreated TZM control; Groups VI: 5 mg/kg/day valsartan +2.25 mg/kg/day TZM; Group VII: 0.25 mg/kg/day amlodipine +2.25 mg/kg/day TZM; Group VIII: 0.035 mg/kg/day lisinopril +2.25 mg/kg/day TZM; Groups IX: [0.25 mg/kg/day amlodipine +0.035 mg/kg/day lisinopril] + 2.25 mg/kg/day TZM; Group X: [5 mg/kg/day valsartan +0.035 mg/kg/day lisinopril] + 2.25 mg/kg/day TZM.

### Amlodipine, Lisinopril, Valsartan and Their Fixed-Dose Combinations on Cardiac Marker Enzymes (*cTnI* and LDH) of *TZM*-Treated Rats

Repeated ADP, LSP, and VAL pretreatments did not significantly (*p* > 0.05) cause alterations in the serum *cTnI* and LDH. However, repeated intraperitoneal *TZM* injections resulted in significant increases (*p* < 0.0001) in the serum *cTnI* and LDH levels when compared to that of untreated negative (control) (Group I) values (Table 6). With oral pretreatments with ADP, LSP, VAL and their fixed-dose combinations [(ADP + LSP) and (VAL + LSP)], increases in the serum *cTnI* and LDL levels were significantly (*p* < 0.05, *p* < 0.001 and *p* < 0.0001) attenuated in *TZM*-intoxicated rats, with the most significant attenuation recorded in rats orally pretreated with the fixed-dose ADP + LSP combination (Table 6). Similar effects were recorded in normal rats orally treated with VAL, ADP, and LSP (Table 6).

**TABLE 6:** Effect of amlodipine, lisinopril, valsartan and their fixed-dose combinations [(ADP + LSP) and (VAL + LSP)] on serum *cTnI* and LDH in *TZM*-intoxicated rats.

Treatment groups	*cTnI* (ng/ml)	LDH (U/L)
I	1.04 ± 0.18	7,521.00 ± 770.40
II	0.87 ± 0.19^c−^	5,620.00 ± 448.70^c−^
III	1.17 ± 0.24^c−^	5,553.00 ± 664.20^c−^
IV	2.27 ± 0.93^c−^	6,187.00 ± 1,046.00^c−^
V	17.09 ± 1.45^c+^	16769.00 ± 2,350.00^c+^
VI	3.93 ± 1.29^b−^	6,771.00 ± 1,586.00^c−^
VII	7.52 ± 2.25^a−^	4,685.00 ± 381.30^c−^
VIII	4.43 ± 1.79^b−^	5,196.00 ± 554.00^c−^
IX	0.87 ± 0.35^c−^	3,694.00 ± 384.30^c−^
X	2.95 ± 1.01^c−^	5,787.00 ± 779.60^c−^

^c+^ represents a significant increase at p < 0.0001 when compared to untreated negative control (Group I) values, while ^a−, b−,^ and ^c−^ represent significant decreases at p < 0.05, p < 0.001, and p < 0.0001, respectively, when compared to untreated TZM-intoxicated (Group V) control values, respectively.

Groups I: untreated normal control; Group II: 5 mg/kg/day valsartan; Group III: 0.25 mg/kg/day amlodipine; Group IV: 0.035 mg/kg/day lisinopril; Group V: untreated TZM control; Groups VI: 5 mg/kg/day valsartan +2.25 mg/kg/day TZM; Group VII: 0.25 mg/kg/day amlodipine +2.25 mg/kg/day TZM; Group VIII: 0.035 mg/kg/day lisinopril +2.25 mg/kg/day TZM; Groups IX: [0.25 mg/kg/day amlodipine +0.035 mg/kg/day lisinopril] + 2.25 mg/kg/day TZM; Group X: [5 mg/kg/day valsartan +0.035 mg/kg/day lisinopril] + 2.25 mg/kg/day TZM.

### Amlodipine, Lisinopril, Valsartan and Their Fixed-Dose combinations on the Cardiac Tissue Oxidative Stress Markers (GSH, GST, GPX, SOD, CAT, and MDA) of *TZM*-Treated Rats

*TZM* intraperitoneal injection to treated rats resulted in significant attenuation (*p* < 0.001 and *p* < 0.0001) in SOD, CAT, GST and GPx activities, and GSH levels, while there were significant increases (*p* < 0.001) in the MDA activities in the treated cardiac tissue (Table 7). However, repeated oral treatments with ADP, LSP, VAL and their fixed-dose combinations [(ADP + LSP) and (VAL + LSP)] significantly (*p* < 0.05, *p* < 0.001, *p* < 0.0001) attenuated the alterations in the activities of these enzyme markers in the cardiac tissue restoring their activities to normal as recorded for Groups I–IV values (Table 7).

**TABLE 7:** Antioxidant activities of amlodipine, lisinopril, valsartan and their fixed-dose combinations in *TZM*-intoxicated cardiac tissue.

Groups	Antioxidant parameters					
	GSH	GST	GPx	SOD	CAT	MDA
I	83.13 ± 5.77	30.80 ± 0.48	93.63 ± 6.18	4.09 ± 0.11	23.02 ± 0.71	1.85 ± 0.31
II	69.83 ± 2.43^a+^	31.67 ± 0.45^b+^	82.07 ± 4.30^b+^	4.44 ± 0.31^c+^	25.28 ± 1.55^b+^	1.87 ± 0.30^c**^
III	76.07 ± 2.62^b+^	36.06 ± 0.43^b+^	89.93 ± 3.81^b+^	4.41 ± 0.31^c+^	26.11 ± 1.09^b +^	1.98 ± 0.28^c**^
IV	92.00 ± 3.77^c+^	40.31 ± 1.81^b+^	102.30 ± 6.99^c+^	4.72 ± 0.30^c+^	26.12 ± 1.40^b +^	1.55 ± 0.24^c**^
V	47.97 ± 1.43^c-^	21.90 ± 2.51^c-^	53.87 ± 2.71^c-^	2.01 ± 0.25^c-^	14.07 ± 1.48^b-^	4.16 ± 0.44^c*^
VI	106.60 ± 15.76^c+^	36.38 ± 0.53^b+^	97.73 ± 2.51^c+^	4.07 ± 0.15^c+^	24.61 ± 1.00^b+^	1.78 ± 0.48^c**^
VII	76.50 ± 2.84^b+^	34.78 ± 0.43^b+^	99.0 ± 1.54^c+^	4.03 ± 0.15^c+^	28.66 ± 1.58^b+^	1.64 ± 0.81^c**^
VIII	89.24 ± 7.32^c+^	35.31 ± 1.63^b+^	100.60 ± 6.91^c+^	4.16 ± 0.24^c+^	24.85 ± 0.80^b+^	1.10 ± 0.15^c**^
IX	71.92 ± 6.61^b+^	31.26 ± 2.28^b+^	72.15 ± 3.16^a+^	4.53 ± 0.24^c+^	23.81 ± 3.88^b+^	2.04 ± 0.52^b**^
X	71.18 ± 3.16^c+^	30.59 ± 2.99^b+^	65.00 ± 6.83^a+^	4.07 ± 0.21^b+^	21.40 ± 4.28^b+^	2.78 ± 0.61^a**^

^b−^ and ^c−^ represent significant decreases at p < 0.001 and p < 0.0001, respectively, when compared to untreated negative control (Group I) values, while ^c*^ represents a significant increase at p < 0.0001 when compared to Group I value; ^a+, b+,^ and ^c+^ represent significant increases at p < 0.05, p < 0.001, and p < 0.0001, respectively, when compared to untreated TZM-intoxicated control (Group V) values, while ^a**, b**,^ and ^c**^ represent significant decreases at p < 0.05, p < 0.001, and p < 0.0001, respectively, when compared to untreated TZM-intoxicated control (Group V) values.

Groups I: untreated normal control; Group II: 5 mg/kg/day valsartan; Group III: 0.25 mg/kg/day amlodipine; Group IV: 0.035 mg/kg/day lisinopril; Group V: untreated TZM control; Groups VI: 5 mg/kg/day valsartan +2.25 mg/kg/day TZM; Group VII: 0.25 mg/kg/day amlodipine +2.25 mg/kg/day TZM; Group VIII: 0.035 mg/kg/day lisinopril +2.25 mg/kg/day TZM; Groups IX: [0.25 mg/kg/day amlodipine +0.035 mg/kg/day lisinopril] + 2.25 mg/kg/day TZM; Group X: [5 mg/kg/day valsartan +0.035 mg/kg/day lisinopril] + 2.25 mg/kg/day TZM.

### Histopathologic Assessment of Drug-Treated Cardiac Tissues

**FIGURE 1:**
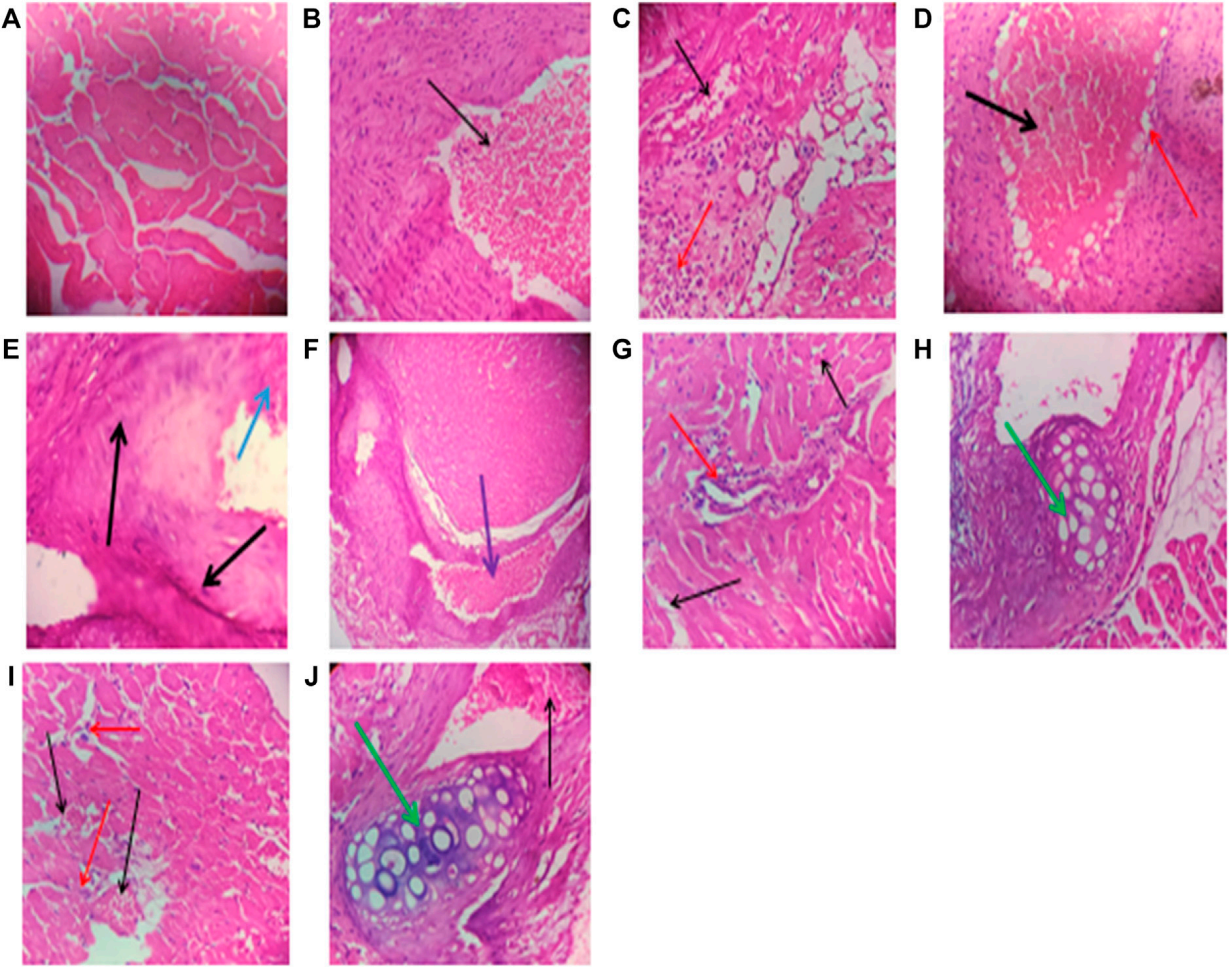
**(A)** A cross-sectional representative of the rat heart tissue pretreated with 10 ml/kg/day/*p.o.* sterile water and 1 ml/kg/day/*i.p.* route of sterile water showing normal cardiac histoarchitecture (×400 magnification, hematoxylin–eosin stain). **(B)** A cross-sectional representative of rat heart pretreated with 5 mg/kg/day/*p.o.* VAL dissolved in sterile water showing mild coronary artery congestion (indicated by the thin black arrow) and normal myocardiocytes (×400 magnification, hematoxylin–eosin stain). **(C)** A cross-sectional representative of the rat heart pretreated with 0.25 mg/kg/day/*p.o.* route ADP dissolved in sterile water showing mild congestion (indicated by the thin black arrow) and lymphocytic infiltration of cardiomyocytes (indicated by the thin red arrow) (×400 magnification, hematoxylin–eosin stain). **(D)** A cross-sectional representative of rat cardiac tissue pretreated with 0.035 mg/kg/day/*p.o.* route LSP dissolved in sterile water showing severe vascular congestion (indicated by the thick black arrow) and diffuse perivascular lymphocytic infiltration (indicated by the thin red arrow) (×400 magnification, hematoxylin–eosin stain). **(E)** A cross-sectional representative of untreated *TZM*-intoxicated rat cardiac tissue pretreated with 10 ml/kg/day/*p.o.* sterile water showing severe vascular and cardiomyocyte congestion (indicated by the thick black arrow) and intraparenchymal hemorrhage (indicated by the thick blue arrow) (×400 magnification, hematoxylin–eosin stain). **(F)** A cross-sectional representative of *TZM*-intoxicated rat cardiac tissue pretreated with 5 mg/kg/day/*p.o.* VAL showing coronary artery recanalization (indicated by the thick purple arrow) (×100 magnification, hematoxylin–eosin stain). **(G)** A cross-sectional representative of *TZM*-intoxicated rat cardiac tissue pretreated with 0.25 mg/kg/day/*p.o.* ADP dissolved in sterile water showing mild cardiac vascular congestion (indicated by the thin black arrow) with sparse lymphocytic infiltration of the cardiomyocytes (indicated by the thin red arrow) (×400 magnification, hematoxylin–eosin stain). **(H)** A cross-sectional representative of the *TZM*-intoxicated cardiac tissue pretreated with 0.035 mg/kg/day/*p.o.* LSP dissolved in sterile water showing moderate vascular congestion and cartilaginous metaplasia within the coronary blood vessel wall (indicated by the thick green arrow) (×400 magnification, hematoxylin–eosin stain). **(I)** A cross-sectional representative of *TZM*-intoxicated rat cardiac tissue pretreated with the fixed dose 0.25 mg/kg/day/*p.o.* route ADP +0.035 mg/kg/day/*p.o.* route LSP combination dissolved in sterile water showing moderate vascular congestion (indicated by the thin black arrow) with sparse lymphocytic infiltration (indicated by the thin red arrow) (×400 magnification, hematoxylin–eosin stain). **(J)** A cross-sectional representative of the *TZM*-intoxicated rat cardiac tissue pretreated with 5 mg/kg/day/*p.o.* route VAL +0.035 mg/kg/day/*p.o.* LSP showing mild-to-moderate vascular congestion (indicated by the thin black arrow) and coronary arterial wall cartilaginous metaplasia within the coronary blood vessel wall (indicated by the thick green arrow) (×400 magnification, hematoxylin–eosin stain).

Repeated intraperitoneal injections of rats with 2.25 mg/kg/day of *TZM* for 7 days resulted in marked vascular and cardiomyocyte congestion, intraparenchymal hemorrhage, and coronary artery microthrombi formation (Figure 5) when compared to normal cardiac histoarchitecture in untreated normal rat heart (Figure 1A). Individual oral pretreatments with VAL and ADP to normal rats did not cause any remarkable histological changes in the cardiac histoarchitecture (Figures 1B and D) although LSP induced severe myocardial congestion and diffuse myocardial lymphocytic infiltration (Figure 1C). However, in *TZM*-intoxicated rats orally pretreated with VAL, there was coronary artery recanalization (Figure 1F), while there were mild congestion with sparse ymphocytic infiltration (Figure 1G) and coronary arterial wall cartilaginous metaplasia (Figure 1H) in ADP and LSP-pretreated cardiac tissues, respectively. Fixed-dose [(ADP + LSP) and (VAL + LSP)] combination pretreatments were associated with mild myocardial congestion (Figure 1I) and coronary arterial wall cartilaginous metaplasia (Figure 1J), respectively.

### Amlodipine, Lisinopril, Valsartan and Their Fixed-Dose Combinations on *TZM*-Treated Cardiac Tissue Caspase-3 and Caspase-9 Levels

**FIGURE2:**
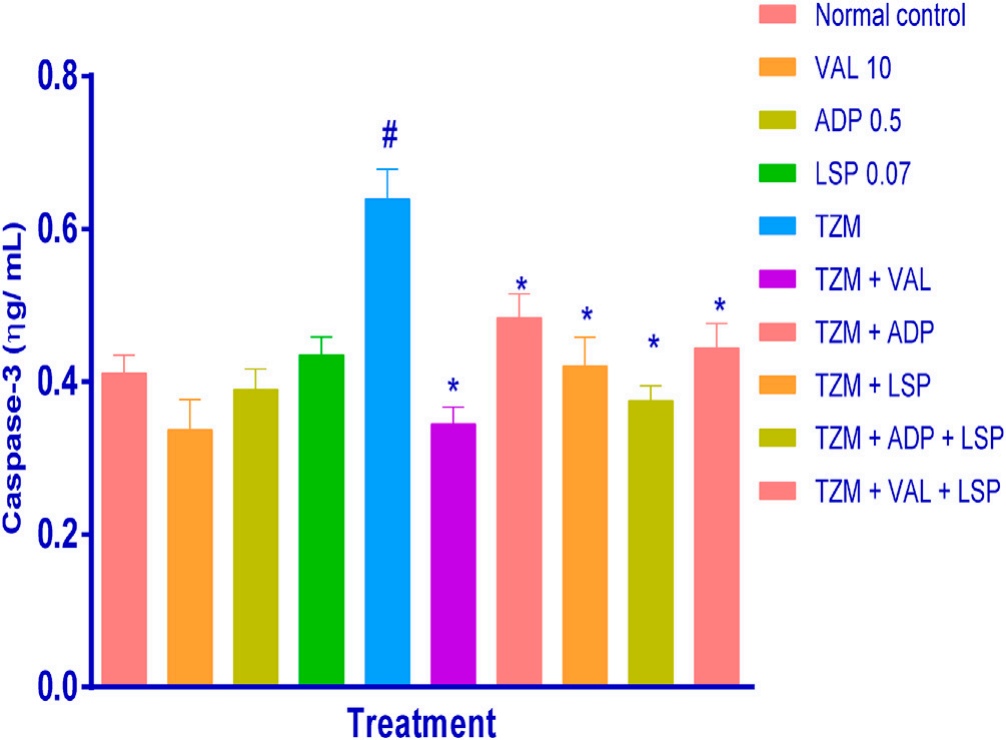
Effect of drug treatment on cardiac tissue caspase-3 levels in rats as measured by ELISA. Bar represents mean ± SEM (*n* = 4), significant difference denoted by #*p* < 0.05 vs. normal control (Group I) or *p < 0.05 vs. *TZM* by One-way ANOVA followed by Turkey’s *post hoc* test. Normal control, VAL-valsartan 5 mg/kg/day, ADP-amlodipine 0.25 mg/kg/day, LSP- lisinopril 0.035 mg/kg/day, and *TZM*-trastuzumab 2.25 mg/kg/day.

Evaluation of the cardiac tissue apoptotic marker level, caspase-3, using ELISA technique showed that caspase-3 was significantly (*p <* 0.05) elevated in untreated *TZM*-treated (Group V) cardiac tissues when compared with the untreated normal control (Group I) (Figure 2). Rats orally pretreated with ADP, LSP, and VAL had no significant alterations in their cardiac caspase-3 levels. However, repeated oral pretreatments with ADP, LSP, VAL and their fixed-dose [(ADP + LSP) and (VAL + LSP)] combinations significantly (*p <* 0.05) attenuated *TZM*-induced elevation in caspase-3 levels in the treated rats (Figure 2).

**FIGURE 3:**
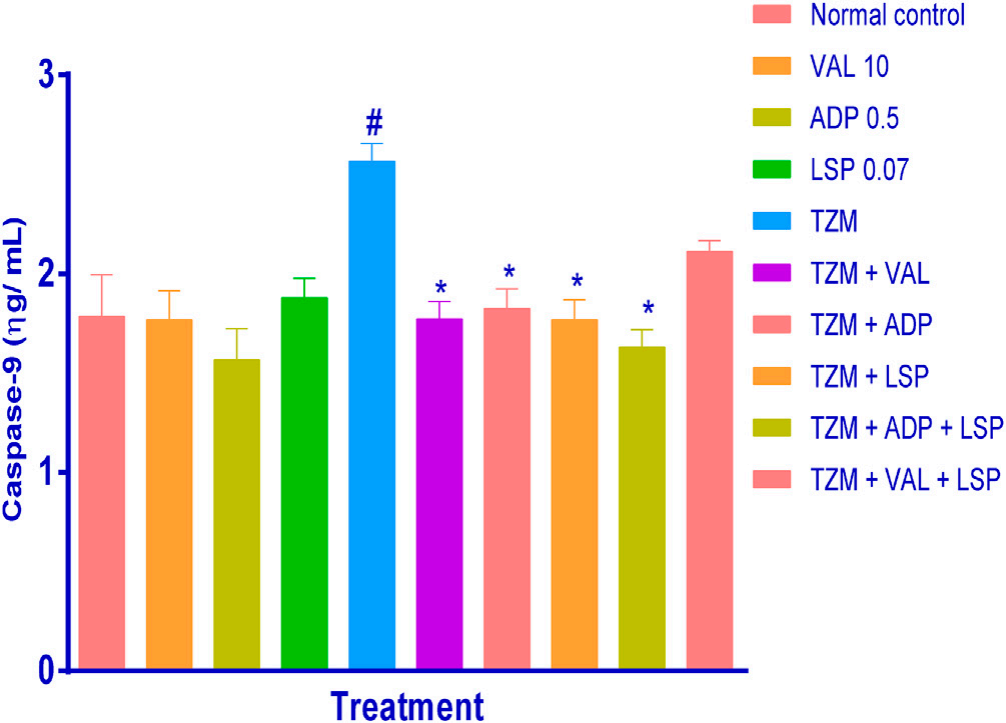
Effect of drug treatment on cardiac tissue caspase-9 levels in rats measured by ELISA. Bar represents mean ± SEM (*n* = 4), significant difference denoted by #*p* < 0.05 vs. normal control or *p < 0.05 vs. *TZM*-only by one-way ANOVA followed by Turkey’s *post hoc* test. Normal control, VAL-valsartan 5 mg/kg/day, ADP-amlodipine 0.25 mg/kg/day, LSP-lisinopril 0.035 mg/kg, and *TZM*-trastuzumab 2.25 mg/kg/day.

Similarly, while oral pretreatments with ADP, LSP and VAL did not induce significant (*p *> 0.05) alterations in the caspase-9 level when compared to untreated normal control group (Figure 3), TZM intoxication (Group V) induced profound (*p *< 0.05) caspase-9 expression in the treated rat cardiac tissue when compared to the untreated normal control (Group I) (Figure 3). However, this increased caspase-9 expression was effectively (*p *< 0.05) attenuated by oral pretreatments with ADP, LSP, VAL and fixed-dose (ADP + LSP) combination except (VAL + LSP) combination which caused an insignificant (*p *> 0.05) reduction in the cardiac tissue caspase-9 expression (Figure 3).

### Amlodipine, Lisinopril, Valsartan and Their Fixed-Dose Combinations on *TZM*-Treated Cardiac Tissue Caspase-3 Expression

**FIGURE 4:**
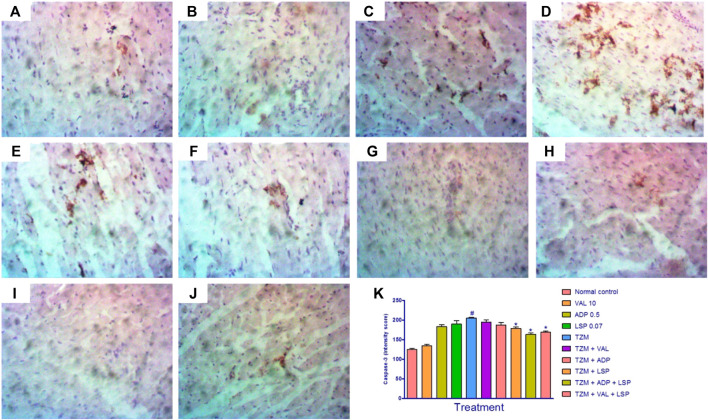
Representative photomicrographs of immunohistochemical expression of caspase-3 in cardiac tissue of rats (magnification ×400). **(A)** Normal control, **(B)** valsartan 10 mg/kg/day, **(C)** amlodipine 0.25 mg/kg/day, **(D)** lisinopril 0.035 mg/kg/day, **(E)** trastuzumab (*TZM*) 2.25 mg/kg/day, **(F)** *TZM* + valsartan 5 mg/kg/day, **(G)** *TZM* + amlodipine 0.25 mg/kg/day, **(H)** *TZM* + lisinopril 0.035 mg/kg, **(I)** *TZM* + amlodipine + lisinopril, **(J)** *TZM* + valsartan + lisinopril, and **(K)** intensity score of caspase-3 expression, mean ± SEM (*n* = 3), and significant difference denoted by #*p* < 0.05 vs. normal control or *p < 0.05 vs. *TZM* by one-way ANOVA followed by Turkey’s *post hoc* test.

Caspase-3 expressions in untreated *TZM*-intoxicated cardiac tissues (Figures 4E) and cardiac tissues pretreated with VAL, ADP, LSP and their fixed-dose [(ADP + LSP) and (VAL + LSP)] combinations are as depicted in Figures 4B–D and F–I, respectively. Untreated *TZM*-intoxicated cardiac tissue showed enhanced expression of caspase-3 (Figures 4E) compared to normal control group (Figures 4A). Quantification of the immunohistochemical intensity showed profound (*p* < 0.05) increase in intensity score suggesting profound enhanced caspase-3 expression in *TZM*-treated rats compared with the untreated normal control (Figure 4K). However, there was a significant (*p* < 0.05) decrease in intensity score of *TZM*-treated rats that were orally pretreated with LSP (Figure 4H) and the fixed-dose [(ADP + LSP) (Figure 4I) and (VAL + LSP) (Figure 4J)] combinations.

### Effect of Amlodipine, Lisinopril, Valsartan and Their Fixed-Dose Combinations on *TZM*-Treated Cardiac Tissue BCL-2 Expression

**FIGURE 5:**
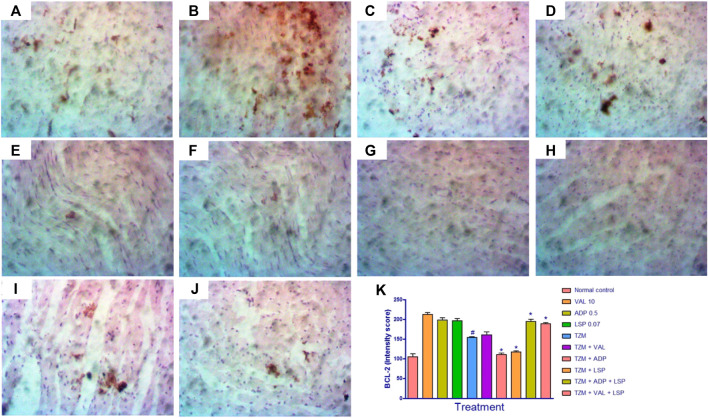
Representative photomicrographs of immunohistochemical expression of BCL-2 in cardiac tissue of rats (magnification ×400). **(A)** Normal control, **(B)** valsartan (VAL) 5 mg/kg/day, **(C)** amlodipine (ADP) 0.5 mg/kg/day, **(D)** lisinopril (LSP) 0.035 mg/kg/day, **(E)** trastuzumab (*TZM*) 2.25 mg/kg/day, **(F)** *TZM* + valsartan 5 mg/kg/day, **(G)** *TZM* + amlodipine 0.25 mg/kg/day, **(H)** *TZM* + lisinopril 0.035 mg/kg/day, **(I)** *TZM* + amlodipine + lisinopril, **(J)** *TZM* + valsartan + lisinopril, and **(K)** intensity score of BCL-2 expression, mean ± SEM (*n* = 3), and significant difference denoted by ^#^ *p* < 0.05 vs. normal control or *p < 0.05 vs*. TZM* by one-way ANOVA followed by Turkey’s *post hoc* test.

The photomicrographs of immunohistochemical staining for BCL-2 expression in cardiac tissues are as shown in Figures 5A–J. While normal rats did not show significant BCL-2 expression (Figures 5A,K), there was a significant (*p* < 0.0) reduction in the BCL-2 expression in the untreated *TZM*-intoxicated rats as indicated in by intensity scores (Figures 5E,K). Similarly, significant (*p* < 0.05) reductions in BCL2 expressions were recorded for rats orally pretreated with ADP (Figures 5G,K) and LSP (Figures 5H,K). However, there was a significant (*p* < 0.05) increase in the BCL2 expression in rats orally pretreated with fixed-dose [(ADP + LSP) (Figures 5I,K) and (VAL + LSP) (Figures 5J,K) combinations (Figures 5E,K).

## Discussion

The monoclonal antibody, trastuzumab, not only to improve treatment outcome but also remains the gold standard in the treatment of locoregional and advanced overexpressing HER2 breast cancer with its most notorious off-target side effect being cardiotoxicity since HER2 is involved in myocardial homeostasis (Tocchetti et al., 2012; Hamed et al., 2016). Clinically, *TZM*-induced cardiotoxicity may manifest as left ventricular dysfunction (Tocchetti et al., 2012), hypertensive crisis (Herrmann et al., 2014), which can decompensate to heart failure, and ultimately cardiac death (Bowles, 2012; Ezaz et al., 2014; Cuomo et al., 2019). Although the exact mechanism(s) of *TZM*-mediated cardiotoxicity is primarily type II chemotheraphy-related cardiotoxicity (being dose-independent, largely reversible, and does not produce ultrastructural changes on histological examination) (Hamed et al., 2016) but has been proposed to result from a “dual-hit” mechanism namely: direct inhibition of antiapoptotic pathways and upregulation of angiotensin II which induces apoptosis through AT_1_ receptor as well activation of NADPH oxidase leading to cell death through mitochondrial dysfunction (Spector and Blackwell, 2009; Zeglinski et al., 2011; Sadek et al., 2017), with resultant increase in reactive oxygen species (ROS) production and inhibition of neuregulin-1 (NRG-1) signaling pathway (Hamed et al., 2016; Sadek et al., 2017). *TZM* cardiotoxicity is also believed to be related to antibody-dependent and complement-dependent cytotoxicity (Force et al., 2007).

Previous studies have reported the protective role that some classes of antihypertensive agents play in ameliorating *TZM*- and anthracycline-induced cardiotoxicity (Hahn et al., 2014; Rygiel, 2016; Brown et al., 2020). These classes include cardioselective β_1_-adrenoceptor blockers ( bisoprolol, carvedilol, metoprolol, *etc*) (Nohria, 2013; Pituskin et al., 2017; Brown et al., 2020), calcium channel blockers (e.g., amlodipine), angiotensin-converting enzyme inhibitors ( lisinopril, enalapril, etc.) (Guglin et al., 2019; Vaduganathan et al., 2019; Brown et al., 2020), and angiotensin receptor blockers (ARBs) (losartan, valsartan, telmisartan, candesartan, *etc*) (Boekhout et al., 2016; Gulati et al., 2016), although there have been conflicting results of the therapeutic benefits of these antihypertensive classes of drugs (Gujral et al., 2018). In view of these drawbacks, the present study aimed at evaluating the therapeutic potentials of amlodipine, lisinopril, valsartan individually and their fixed-dose combinations in ameliorating *TZM*-associated cardiotoxicity in experimental rats using biochemical (cardiac enzyme markers, complete lipids profile, cardiac oxidative stress markers, and apoptosis markers), histopathological, and immuhistochemical endpoints.

*TZM* treatment is known to cause the type of cardiac injury that is characterized by elevated serum cardiac specific biomarkers such as cardiac troponins I and T (*cTnI* and *cTnT*), LDH, brain (B-type) natriuretic peptide (BNP), CK-MB (Singh et al., 2015; Ananthan and Lyon, 2020; Demissei et al., 2020), and pro-inflammatory mediators such as interleukin (IL)-6, c-reactive protein, myeloperoxidase, galectin-3, and growth differentiation factor-15 (Ananthan and Lyon, 2020; Demissei et al., 2020). In this study, *TZM*-induced cardiotoxicity was marked by profound elevation in the serum *cTnI* and LDL, and these elevations were reliably attenuated by ADP, LSP, VAL and their fixed-dose [(ADP + LSP) and (VAL + LSP)] combinations indicating cardioprotective potential of these drugs in *TZM*-mediated cardiotoxicity. Previous studies, however, have shown LSP and carvedilol to profoundly protect against *TZM*-mediated serum elevation of these cardiac enzyme markers (Witteles and Bosch, 2015; Guglin et al., 2019). Similar protective effect was reportedly offered by VAL in breast cancer patients on trastuzumab chemotherapy (Al-Hamadi et al., 2018) as well as ADP (Keefe, 2002; Jones et al., 2009; Thomas, 2017), thus, lending support to our findings.

The renin–angiotensin system (RAS) plays a critical role in the development of myocardiac hypertrophy, cardiac failure, and reperfusion injury (Zablocki and Sadoshima 2013; Pinter et al., 2018) and its suppression by angiotensin-converting enzyme inhibitors equally ameliorate heart remodeling process, thereby, prolonging long-term survival time in animal models and humans with cardiac hypertrophy, failure, and reperfusion injury (Iqbal et al., 2008; Akolkar et al., 2015). At the molecular level, angiotensin II effectively down-regulates the actions of the NRG-1/ErB system (Lemmens et al., 2006), suggesting that the beneficial role of angiotensin-converting enzyme (ACE) inhibition may be related to this effect (Cardinale et al., 2006; Munster et al., 2019). Thus, lisinopril, could be mediating its cardioprotective mechanism in *TZM* cardiotoxicity *via* the heart remodeling pathway.

Oxidative and nitrative stress have been implicated in the pathophysiology of *TZM*-mediated cardiotoxicity through generation of highly toxic reactive oxygen species (ROS) and nitrative species by impeding HER-2 signaling and inhibiting tissue pro-survival effects (Deavali et al., 2012; Mohan et al., 2017; Gorini et al., 2018). *TZM* is known to interfere with mitochondrial functionality to cause mitochondrial dysfunction, ATP depletion, and inhibiting AMPK and PI3K/Akt pathways (Teppo et al., 2017; Gorini et al., 2018). *TZM* sets off proapoptotic pathway proteins such as *Bax* and can trigger the opening of *mPTP*, consequently resulting in mitochondrial dysfunction and ROS accumulation (Gordon et al., 2009). Similarly, *TZM* also binds to HER-2 and increases proapoptotic Bcl-xS expression while it decreases antiapoptotic *Bcl-xL* expression (Grazette et al., 2004; Sadek et al., 2017). These result in overwhelming ROS production and reduced ROS scavenging activities with consequent profound inhibition of SOD, CAT, GST, and GPx activities, reduced GSH levels as well as increased MDA levels of *TZM*-treated tissues (Dirican et al., 2014). The fact that the referred cardiac oxidative stress enzyme activities were profoundly inhibited by the *TZM* treatment, our finding is, thus, in tandem with other earlier findings (Dirican et al., 2014; Teppo et al., 2017; Gorini et al., 2018). Also, the fact that VAL, LSP, ADP and their fixed-dose combinations profoundly improved the activities of oxidative stress marker enzymes in the treated rats strongly suggest the protective role of these antihypertensive agents in *TZM*-induced tissue oxidative stress mediated *via* reduced caspase-3 and caspase-9 production as well as increased antioxidant mechanisms.

Another significant finding of this study is the effect of ADP, LSP, VAL and their fixed-dose combination pretreatments and *TZM* treatments on serum complete lipid profile. In this study, the fact that repeated *TZM* treatment was associated with resultant marked hypertriglyceridemia without corresponding hypercholesterolemia is a strong indication that *TZM* could increase the risk for an adverse cardiovascular event such as angina pectoris, ischemic heart disease, cardiomyopathy, stroke, and heart failure. This assertion is in consonance with previous studies that have reported *TZM* to increase the risk for coronary artery disease and ischemic heart disease (Rushton et al., 2017; Yuan et al., 2018; Montemurro et al., 2019; Chaturvedi et al., 2020). The fact that ADP, LSP, and VAL pretreatment individually attenuated *TZM*-associated hypertriglyceridemia is notable and indicate cardioprotective potentials of these drugs although their fixed-dose combinations worsened *TZM*’s potentials for dyslipidemia and increased risk for cardiovascular events. The reason and underlying mechanism(s) for these observations are beyond the scope and cannot be deduced from results of the current study but would be worth exploring in the nearest future.

Atherogenic index of plasma (AIP) is a very reliably crucial index that can be used as a stand-alone index for cardiac risk estimation and changes in the levels of any lipid profile predisposes to atherosclerosis and its associated complication (Kanthe et al., 2012; Khazaal, 2013). It is defined as logarithm [log_10_] of the ratio of plasma concentration of TG to HDL-c and is strongly correlated with cardiovascular risks and as such could be considered an adjunct over the individual lipid profile (Dobiasova, 2004; Nansseu et al., 2016). AIP is, thus, considered the best determinant for fractionated esterification rate of HDL-c and more useful than routine lipid parameters (Bo et al., 2018). The AIP value of <0.11 is considered low risk, while AIP values of 0.11–0.21 and >0.21 are considered intermediate risk and increased risk, respectively. The fact that the AIP estimates for the fixed-dose combinations of ADP + LSP and VAL + LSP were higher than 0.21, strongly indicates that these fixed-dose combinations could further increase the risk for *TZM*-induced atherosclerosis although previous studies have reported *TZM* to be atherogenic in nature (Yu et al., 2015; Di Lisi et al., 2017; Yersal et al., 2018). Conversely, the fact that VAL, ADP, and LSP profoundly decreased the AIP value to less than 0.11 is another strong indication that these drugs individually could be attenuating *TZM*-induced atherosclerosis. This finding is, thus, in complete agreement with an earlier study that reported amlodipine and valsartan to improve AIP of patients with end-stage chronic renal disease on hemodialysis and peritoneal dialysis (Erdur et al., 2013).

The effect of *TZM* on weight gain pattern is also significant. Repeated *TZM* treatment was associated with profound weight loss. Again, this finding is in tandem with previous studies that have reported similar weight loss in patients on *TZM* treatment (Hajjaji et al., 2014) although the *TZM* has been reported to have variable effects of body weight pattern in cancer patients on *TZM* treatment (Winkels et al., 2014; Reijers and Burggraaf, 2015; Ginzac et al., 2020). However, oral pretreatment with ADP, LSP, VAL and their fixed-dose combinations further induced weight reduction in the face of *TZM* chemotherapy highlighting the weight loss potential of these antihypertensive drugs and their fixed-dose combinations which were probably mediated *via* plasma norepinephrine, insulin, and leptin inhibitions, and these were previously reported in the obese hypertensive patients on prolonged amlodipine, lisinopril, valsartan and fixed-dose amlodipine–valsartan combination use (Masuo et al., 2001; Villecco et al., 2004).

*TZM* is known to cause related cardiac dysfunction without corresponding histoarchitectural distortion of the myocytes (Jones et al., 2009) although *TZM* was recently reported to induce severe vascular congestion and associated microthrombi formation (Olorundare et al., 2020) in treated experimental rats which the present study is in tandem with. However, the fact that these histopathological changes were profoundly improved by ADP, VAL, and fixed-dose (ADP + VAL) combination pretreatments strongly suggests the cardioprotective potential of these drugs. Another recognizable finding of this study is the histological finding of coronary artery cartilaginous metaplasia which was a prominent cardiac histopathological feature found in rat hearts pretreated with LSP- and fixed-dose VAL–LSP combination. Vascular cartilaginous/osseous metaplasia, which is often associated with the presence of arterial chondrocytes that express type II collagen, is known to be part of the progression of mineralization or atherosclerotic lesion (Fitzpatrick et al., 2003; Nguyen et al., 2012). It also provides evidence of cardiac extracellular matrix remodeling for post-infarcted heart and may constitute a supplemental factor for heart failure when it calcifies (Manole et al., 2019; Carreon et al., 2020). Cartilaginous metaplasia is considered a potential great risk for arterial wall calcification associated with the atherosclerotic plaque (Qiao et al., 1995). Thus, the marked presence of coronary artery cartilaginous metaplasia as seen in cardiac tissues treated with the fixed-dose valsartan–lisinopril (VAL + LSP) combination strongly suggests either vascular remodeling of the *TZM*-mediated endothelial injury or coronary artery atheromatous plaque formation. However, the latter appears to be more likely as the histological finding of coronary artery cartilaginous metaplasia was corroborated by the profound increases in the AI and CRI values. This finding, thus, suggests that the fixed-dose valsartan–lisinopril combination could likely increase the risk for coronary artery atheromatous plaque formation in patients on *TZM* treatment.

In conclusion, findings of this study highlight the promising therapeutic potentials of the antihypertensives–amlodipine, lisinopril, valsartan, and their fixed-dose combinations as repurposed therapeutics in the management of *TZM*-induced cardiotoxicity, partly mediated *via* antiapoptotic and oxidative stress inhibition mechanisms. However, lisinopril and fixed-dose valsartan–lisinopril combination should be used with caution in cancer patients already on trastuzumab therapy as they could predispose to coronary artery atheromatous plaque formation.

## Data Availability

The raw data supporting the conclusions of this article will be made available by the authors, without undue reservation.

## Glossary

ACE: angiotensin-converting enzyme
ACEIs: angiotensin-converting enzyme inhibitors
ADP: amlodipine
AIP: atherogenic index of plasma
ALB: albumin
AMPK: 5′ adenosine monophosphate-activated protein kinase
ANOVA: Analysis of variance
ARBs: angiotensin receptor blockers
AT1: angiotensin II receptor type 1
ATP: adenosine triphosphate
*Bax*: Bcl-2 associated x protein
BCL-2: B-cell lymphoma
Bcl-xL: B-cell lymphoma-extra large
Bcl-xS: B-cell lymphoma-extra small
BNP: brain (B-type) natriuretic peptide
CAT: catalase
CK-MB: creatine kinase-MB
CRI: coronary artery index
*cTnI*: cardiac troponin I
*cTnT*: cardiac troponin T
DAB: 3,3′ diaminobenzidine
DPPH: 1,1-diphenyl-2-picrylhydrazyl
DPX: Dibutylphthalate polystyrene
DTNB: 5,5-dithiobisnitro benzoic acid
ELISA: Enzyme-linked immunosorbent assay
ErbB2: human erythroblastic leukemic viral oncogene B2
ET-1: endothelin-1
GPx: glutathione peroxidase
GSH: reduced glutathione
GST: glutathione S-transferase
H_2_O_2_: hydrogen peroxide
HCl: hydrochloric acid
HDL-c: high density lipoprotein cholesterol fraction
HER-2: human epidermal growth factor receptor-2
IHC: immunohistochemistry
*i.p.*: intraperitoneal
KCl: potassium chloride
LASUCOM: Lagos State University College of Medicine
LDH: lactate dehydrogenase
LDL-c: low density lipoprotein cholesterol fraction
LSP: lisinopril
MDA: malondialdehyde
MAPK: mitogen activated protein kinase
*mPTP*: mitochondrial permeability transition pore
NADPH: nicotinamide adenine dinucleotide phosphate
NO/cGMP: nitric oxide–cyclic guanosine monophosphate
NRG-1/ErbB: neuregulin-1/erythroblastic leukemia viral oncogene
PI3k/Akt: phosphatidylinositol 3-kinase/protein kinase B
p.o.: per os
% ∆bwt.: percentage change in weight
RAS: renin angiotensin system
RKW: relative kidney weight
RLW: relative liver weight
ROS: reactive oxygen species
S.D.: standard deviation
S.E.M.: standard error of the mean
SOD: superoxidase dismutase
TBA: thiobarbituric acid
TC: total cholesterol
TCA: tricarboxylic acid
TG: triglyceride
TP: total protein
*TZM*: trastuzumab (r-DNA origin)
UERC: University Ethics and Research Committee
UNILORIN: University of Ilorin
UV: ultraviolet
VAL: valsartan
VLDL-c: very low density lipoprotein cholesterol fraction
XO: xylenol orange
